# Interpretation of seizure evolution pathways via a mean-field cortical model

**DOI:** 10.1186/1471-2202-13-S1-P95

**Published:** 2012-07-16

**Authors:** Vera M Dadok, Andrew J Szeri, Heidi Kirsch, Jamie Sleigh, Beth Lopour

**Affiliations:** 1 Department of Mechanical Engineering, University of California, Berkeley, CA 94720, USA; 2 Center for Neural Engineering and Prostheses, UC Berkeley and UC San Francisco, CA, USA; 3 School of Medicine, University of California, San Francisco, CA, 94143, USA; 4 School of Medicine, University of Auckland, Grafton, Auckland, 1142, New Zealand; 5 Department of Neurobiology, University of California, Los Angeles, CA, 90095, USA

## 

Treatment of epilepsy is a challenging task. Difficulties arise in choosing the best pharmaceutical drugs for specific patients, and if limited benefits result, attempting to choose the best alternative treatment. It is not always clear why a treatment that works well for one patient does not work well for another. Better understanding of the epileptic brain, such as differentiating between possible biological mechanisms driving seizure evolution, may offer insight into these problems.

**Figure 1:**
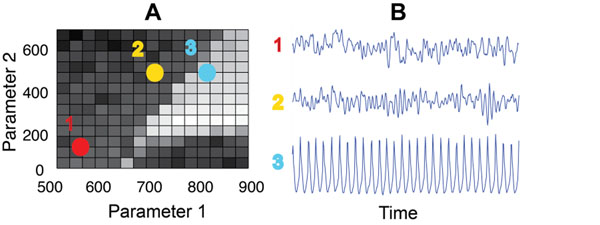
Illustrates a possible path via two model parameters varying and the resultant ECoG-like data. The exact definitions of all parameters are left to the poster. In this illustrative figure parameter 1 is related to the number of inhibitory synaptic connections and parameter 2 is related to the strength of the subcortical input on excitatory neurons in the cortex. **A.** Locations in parameter space. Shading relates to the permutation complexity of the signal. **B.** ECoG-like data signals generated by the model corresponding to locations in parameter space. At locations 1 and 2, the simulated signal through time shows normal cortex activity. At location 3, the ECoG-like data signal through time is in a seizure state with more regular sustained oscillations.

This work makes use of a dynamical model of the human cortex based on the underlying physiology and local anatomy of the brain [1]. This mean-field model can generate electrocorticogram-like (ECoG-like) data in both normal states and seizing states under different plausible parameter configurations [2,3]. There are several different sets of parameters which, when changed, drive the model from a non-seizing state into a seizing state and back. An example of one such pathway is displayed in Figure 1. The presence of different parameter sets that can trigger seizures or exit seizures when varied indicates that there are different biological pathways to and from seizure, and these pathways can be described in terms of the fundamental physical quantities in the model [2]. This work will demonstrate a method that takes ECoG-like data from a model cortex evolving into seizure and identifies which parameters are changing—thus identifying which associated physiological mechanisms may be leading the brain into a seizure. In conjunction with other tools, this method will leverage feature selection and dimensionality reduction algorithms. The same technique may be applied to experimentally collected ECoG seizure data as well.
